# Substrate Modulation of Fatty Acid Effects on Energization and Respiration of Kidney Proximal Tubules during Hypoxia/Reoxygenation

**DOI:** 10.1371/journal.pone.0094584

**Published:** 2014-04-11

**Authors:** Anja Bienholz, Ahmad Al-Taweel, Nancy F. Roeser, Andreas Kribben, Thorsten Feldkamp, Joel M. Weinberg

**Affiliations:** 1 Division of Nephrology, Department of Internal Medicine, Veterans Affairs Ann Arbor Healthcare System and University of Michigan, Ann Arbor, Michigan, United States of America; 2 Division of Nephrology, Department of Internal Medicine, University Duisburg-Essen, Essen, Germany; 3 Division of Nephrology and Hypertension, Department of Internal Medicine, Christian-Albrechts-University, Kiel, Germany; Universidad Pablo de Olavide, Centro Andaluz de Biología del Desarrollo-CSIC, Spain

## Abstract

Kidney proximal tubules subjected to hypoxia/reoxygenation develop a nonesterified fatty acid-induced energetic deficit characterized by persistent partial mitochondrial deenergization that can be prevented and reversed by citric acid cycle substrates. To further assess the role of competition between fatty acids and substrates on inner membrane substrate carriers in the deenergization and the contribution to deenergization of fatty acid effects on respiratory function, digitonin-permeabilized rabbit and mouse tubules were studied using either addition of exogenous oleate after control normoxic incubation or increases of endogenous fatty acids produced by hypoxia/reoxygenation. The results demonstrated major effects of matrix oxaloacetate accumulation on succinate-supported energization and respiration and their modification by fatty acids. Improvements of energization in the presence of fatty acids by glutamate were shown to result predominantly from lowering matrix oxaloacetate rather than from amelioration of transmembrane cycling of fatty acids and uncoupling. Mouse tubules had 2.5 fold higher rates of succinate utilization, which resulted in stronger effects of oxaloacetate accumulation than rabbit tubules. Hypoxia/reoxygenation induced respiratory inhibition that was more severe for complex I-dependent substrates. Fatty acids themselves did not acutely contribute to this respiratory inhibition, but lowering them during 60 min. reoxygenation to allow recovery of ATP during that period alleviated it. These data clarify the basis for the nonesterified fatty acid-induced mitochondrial energetic deficit in kidney proximal tubules that impairs structural and functional recovery and provide insight into interactions that need to be considered in the design of substrate-based interventions to improve mitochondrial function.

## Introduction

Mitochondria of kidney proximal tubules figure prominently in the development of acute kidney injury via their contributions to compromised energetics [1]–[3], by generation of reactive oxygen species that induce both damaging and protective events including sustained upregulation of proinflammatory processes [4], as central mediators of both the intrinsic and extrinsic pathways of apoptosis [5], and as targets of autophagy [6]. Recent observations during controlled clinical ischemia/reperfusion illustrate their involvement in human acute kidney injury [7].

When freshly isolated kidney proximal tubules are subjected to hypoxia/reoxygenation (H/R) ex vivo they develop a reversible energetic deficit characterized by persistent ATP depletion [1], [2], [8] due to mitochondrial deenergization caused by nonesterified fatty acid (NEFA) accumulation [9]. The energetic deficit profoundly impairs their ability to recover structure and function [1], [3]. Moreover, persistent elevation of NEFA and failure to reverse the energetic deficit can lead to further damaging events such as development of the mitochondrial permeability transition [10], [11]. NEFA are well established contributors to both acute kidney injury and chronic kidney disease in vivo [12]–[14].

The energetic deficit can be prevented and reversed by maneuvers that lower the NEFA burden, including removal of NEFA by binding with delipidated albumin or supplementation with citric acid cycle substrates that can enable anaerobic ATP production to promote re-esterification [2], [8], [9]. Lowering of NEFA by these maneuvers is additive to the NEFA-reducing effects of low pH during hypoxia [9]. The substrates may also ameliorate the deficit by limiting NEFA movement on normal inner mitochondrial membrane anion carriers that can mediate the deenergization by facilitating cycling of NEFA across the inner membrane [15]. Studying NEFA cycling by the anion carries is complicated by the necessary role of the carriers in delivering substrates to the matrix for their metabolism and support of respiration.

Respiration is a central mitochondrial function that can provide considerable insight into mitochondrial physiology and pathophysiology and has been of renewed recent investigative interest in the context of new technology for assessing it [16]. It is intimately linked to the type of metabolic substrate available. Early studies of the energetic deficit indicated that it is paradoxically accompanied by respiratory inhibition rather than by the stimulation expected for uncoupled states [2]. However, there is evidence from work with isolated mitochondria that NEFA can inhibit electron transport under some circumstances [17]. The effects of NEFA on respiration in the tubules and whether they can account for respiratory inhibition during the energetic deficit have not been studied.

Although most studies of the energetic deficit have been done using isolated rabbit tubules, the deficit is fully expressed in the mouse [11]. Available data for the mouse, however, are more limited and a better understanding of similarities and differences between the two types of tubules is of interest given the widespread use of mice for genetic deletion studies of mechanisms of acute kidney injury and the potential for substantial differences in tubule susceptibility to injury between species that could impact on resistance to acute kidney injury and interpretation of the benefits of protective maneuvers being widely studied in them.

The present studies were designed to: 1) further clarify the role of substrate carriers in the NEFA-induced deenergization, 2) assess the respiratory effects of NEFA in the isolated tubules and their contribution to the respiratory alterations observed after H/R, and 3) contrast expression of the energetic deficit in the mouse and the rabbit.

## Materials and Methods

Animal use protocols for the studies in this manuscript adhered to the APS Guiding Principles in the Care and Use of Animals and were approved by the Institutional Animal Care and Use Committee of the University of Michigan (Protocol #09545).

### Chemicals/Materials

Female New Zealand White rabbits (1.5–2.0 kg) were obtained from Harlan (Indianapolis, IN). Male C57/BL6J mice were obtained from The Jackson Laboratory (Bar Harbor, ME). Type I collagenase was from Worthington Biochemical (Lakewood, NJ) and Type F collagenase from Sigma-Aldrich (St. Louis, MO). Percoll was purchased from Amersham Biosciences (Piscataway, NJ). High-purity digitonin (catalog no. 300411) was purchased from Calbiochem (San Diego, CA). All other reagents and chemicals were of the highest available purity and obtained from Sigma-Aldrich, St. Louis, MO. Aqueous stock solutions of experimental reagents were all pH adjusted to pH 7.2. Reagents requiring solubilisation in ethanol were delivered from greater than 300x stock solutions in volumes of ethanol that did not by themselves affect the measured functions.

### Tubule preparation

Rabbits were euthanized with an overdose of ketamine and xylazine, mice were euthanized with isoflurane. Kidneys were either perfused in situ just before removal (rabbits) or injected intraparenchymally immediately after removal (mice) with a cold 95% O_2_/5% CO_2_-gassed solution consisting of 115 mM NaCl, 2.1 mM KCI, 25 mM NaHCO_3_, 1.2 mM KH_2_PO_4_, 2.5 mM CaCl_2_, and 1.2 mM MgCl_2_, 1.2 mM MgS0_4_ 25 mM mannitol, 2.5 mg/ml delipidated bovine serum albumin (dBSA), 5 mM glucose, 4 mM sodium lactate, I mM alanine, and 1 mM sodium butyrate (Solution A) with the addition of 1 mg/ml collagenase (Type I, Worthington Biochemical Corp., Freehold). The cortices were then dissected and minced on an ice cold tile, then resuspended in additional Solution A for 8–10 min. of digestion at 37°C followed by enrichment of proximal tubules using centrifugation on self-forming Percoll gradients as previously described [9]–[11], [18], [19].

### Experimental procedures for isolated tubules [9]–[11], [18], [19]

Tubules were suspended at 1.0–2.0 mg tubule protein/ml in a 95% air/5% CO_2_-gassed medium containing 110 mM NaCl, 2.6 mM KCl, 25 mM NaHCO_3_, 2.4 mM KH_2_PO_4_, 1.25 mM CaCl_2_, 1.2 mM MgCl_2_, 1.2 mM MgSO_4_, 5 mM glucose, 4 mM sodium lactate, 0.3 mM alanine, 5 mM sodium butyrate, 2 mM glycine, and 1.0 mg/ml bovine gelatin (75 bloom) (Solution B). For studies limited to normoxic conditions, tubules were preincubated for 15 min. at 37°C, then were resuspended in fresh Solution B containing 2 mM heptanoic acid instead of sodium butyrate for the desired duration. For studies comparing normoxia with H/R, at the end of the 15 min. preincubation tubules were resuspended in fresh Solution B and regassed with either 95% air/5% CO_2_ (normoxic controls) or 95% N_2_/5% CO_2_ (hypoxia). The N_2_/CO_2_ was certified to have <5 ppm O_2_ contamination. Tubule preparations such as ours have been shown to develop significant hypoxia when O_2_ concentrations are <1% [20], [21]. Their ATP concentrations rapidly decrease to <5% of normal under the hypoxia gassing conditions used and this produces severe cell injury and extensive death in the absence of protective maneuvers [1], [2], [8], [11]. During hypoxia, Solution B was kept at pH 6.9 to simulate tissue acidosis during ischemia in vivo [22] and the usual substrates, glucose, lactate, alanine, and butyrate, were omitted. Durations of hypoxia for each of the species, 30 min. (mouse) and 67.5 min. (rabbit), were chosen based on prior time course studies showing that they were optimal for fully expressing the NEFA-mediated energetic deficit. We have previously reported those time course data fully for the rabbit [1], and on the full profile of behavior at those time points in both species [1]–[3], [8]–[11], [15], [19], [23]–[25]. At the end of hypoxia, samples were removed for analysis. The remaining tubules were pelleted and then resuspended in fresh 95% air/5% CO_2_-gassed, pH 7.4 Solution B with experimental agents as needed. Sodium butyrate in Solution B was replaced with 2 mM heptanoic acid during reoxygenation, and, to assure availability of purine precursors for ATP resynthesis, 250 µM AMP was included. After 60 min. of reoxygenation, tubules were sampled for assays.

### Assessment of ΔΨ_m_ using safranin O [9]–[11], [19]

At the end of the desired experimental period samples of tubule suspension were immediately diluted into ice-cold Solution C supplemented with 2.0 mg/ml bovine gelatin, washed once in the same solution, and then held in it at 4°C until use. For the safranin O uptake measurements, the tubules in the holding solution were pelleted and resuspended at a final concentration of 0.10–0.15 mg/ml in an intracellular buffer type solution containing 120 mM KCl, 1 mM KH_2_PO_4_, 1 mM EGTA, 5 µM safranin O, 100–150 µg digitonin/mg protein, and 10 mM K-HEPES, pH 7.2 at 37°C (Solution D) supplemented with 4 mM concentrations of potassium salts of the substrates to be tested. Additional experimental agents were added as indicated with specific experiments. Fluorescence was measured once every second at 485 nm excitation, 586 nm emission using Photon Technology International (Lawrenceville, N.J.) Deltascan, Alphascan, and Quantascan fluorometers, equipped with temperature controlled (37°C), magnetically stirred cuvette holders to follow safranin O uptake by the mitochondria.

### Tubule respiration

Respiration was measured with a Clark oxygen electrode in a 2.5 ml, sealed, temperature-controlled chamber equipped with a magnetic stirrer (Oxygraph System, Hansatech Instruments Ltd., Norfolk, UK). Tubules were resuspended at a final concentration of 0.2–0.6 mg/ml in solution D as used for safranin O uptake studies. Depending on conditions needed, experimental additions included: 4 mM concentrations potassium salts of glutamate, α-ketoglutarate (αKG), malate and succinate, 0.5 mg/ml dBSA, 0.5 mM ADP+4 mM sodium phosphate, 1 µg/ml oligomycin, carbonylcycanide-m-chlorphenylhydrazone (CCCP), 1 µM rotenone. Because CCCP inhibited respiration at excess concentrations and the maximally effective concentration varied for each type of tubule and experimental condition, amounts used were optimized for each setting and ranged from 0.05–0.3 µM without dBSA and 0.3–0.5 µM with dBSA.

### Statistics

Paired and unpaired t-tests are used as appropriate. Where experiments consisted of multiple groups they were analyzed statistically by analysis of variance for repeated measure or independent group designs as needed. Individual group comparisons for the multi-group studies were then made using the Holm-Sidak test for multiple comparisons (SigmaStat 3, SPSS, Chicago, IL). P<0.05 was considered to be statistically significant.

## Results

### Effects of substrate type on response of energization and respiration to oleate

**Figure 1:**
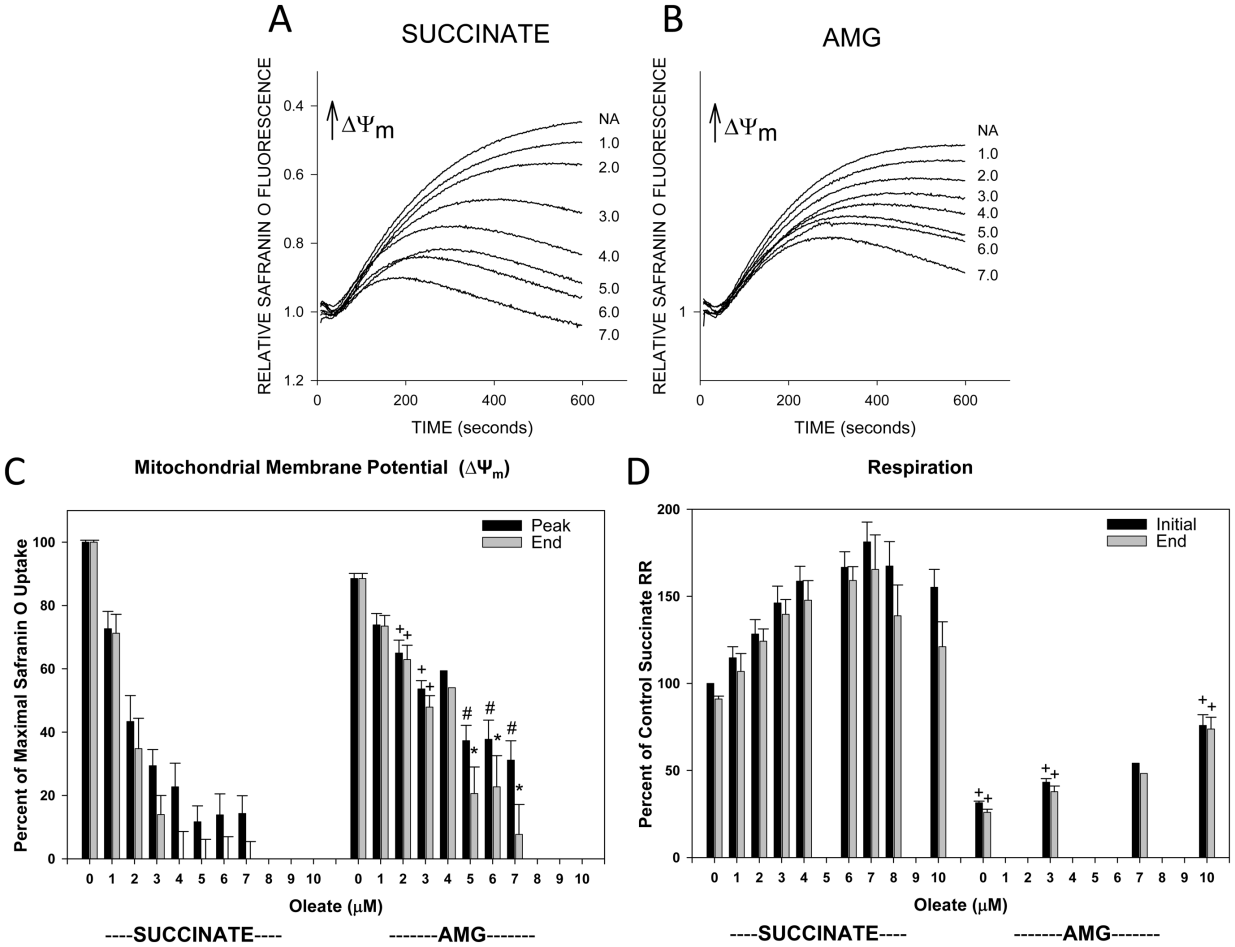
Effects of oleate on energization and respiration of permeabilized tubules: comparison of complex I and complex II-dependent substrates. Direct effects of oleate on energization of permeabilized rabbit tubules measured with safranin O uptake (panels A–C) and respiration (D) supported by either succinate or the combination of complex I dependent substrates, α-ketoglutarate, malate, and glutamate (AMG). Sets of typical safranin O uptake tracings (inverted fluorescence) are shown in panels A and B and group averages for those studies are in panel C. Numbers adjacent to each tracing in panels A are the concentrations of oleate added in µM. In panel C, “Peak” indicates the maximal uptake compared to the uptake seen without added oleate using succinate as substrate measured in the presence of delipidated albumin to eliminate the effect of endogenous fatty acids. “End” indicates the final level reached at the end of the 600 second measurement period, which can be less than the peak if there has been decay of ΔΨm. The panel D respiratory rates (RR) are given as percentages relative to “Control” rates without added oleate using succinate as substrate. Shown are both the initial rate produced by oleate and then the rate at the end of 600 seconds of measurement. Values in panels C and D are means±SEM for N = 3–5 except 4 µM oleate with AMG where the N was 2. *P<0.05, #P<0.01, +P<0.001 vs. corresponding succinate group.

Fig. 1 summarizes the effects of oleate on ΔΨ_m_ measured as safranin O uptake and respiration of normoxic rabbit tubules. Progressive deenergization occurred as the oleate concentration was increased from 1 µM up to 7 µM, the concentration where energization in the presence of the complex II-dependent substrate, succinate, was completely blocked by the end of the 600 second measurement period (Figs. 1A and 1C). Although ΔΨ_m_ in the absence of added oleate was slightly lower with the complex I-dependent substrate combination, αKG+malate+glutamate (AMG), sensitivity to oleate-induced deenergization was less with AMG than with succinate at all concentrations >1.0 µM (Figs. 1B and 1C). With succinate as substrate, oleate stimulated respiration at concentrations up to 7 µM, but at higher concentrations of oleate, the rates at the end of the measurement period began to decay and the initial rates were less strongly stimulated (Fig. 1D). The basal respiratory rate supported by AMG in the absence of oleate was much lower than with succinate, but progressive stimulation was seen at all oleate concentrations up to the 10 µM maximum that was tested (Fig. 1D). Taking into account the amount of protein used, addition of 3 uM oleate as done in these experiments delivers 20 nmol/mg protein, which is similar to the 20–30 nmol/mg protein levels measured at the end of hypoxia in intact isolated tubules [9].

### Effects of additional substrates on NEFA-induced deenergization

**Figure 2:**
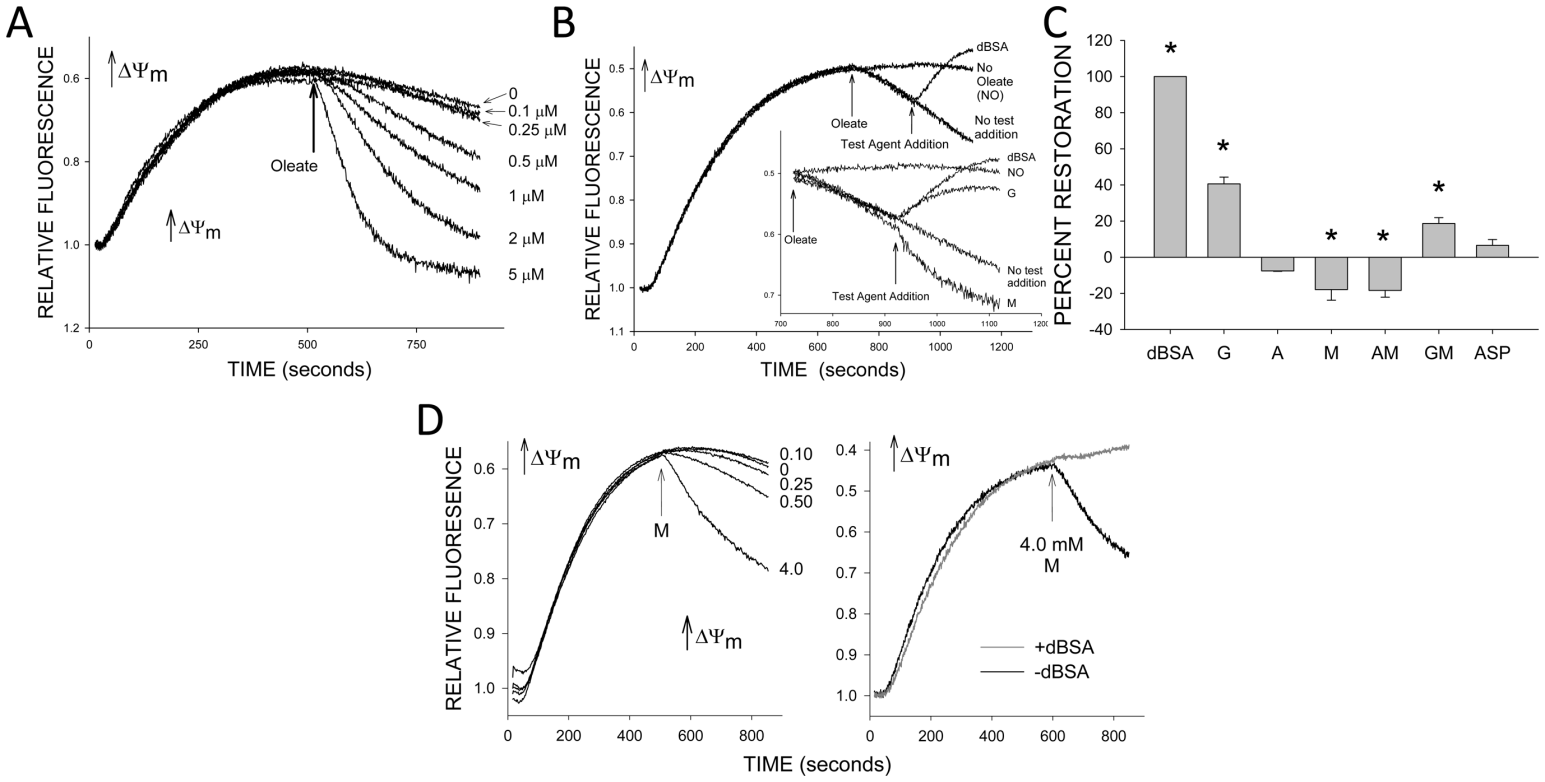
Effects of additional substrates on oleate-induced deenergization in the presence of succinate. A. Concentration dependence of deenergization produced by late addition of oleate to rabbit tubules. B. Representative experiment showing how modification of late deenergization by oleate was tested. 1 µM oleate was added at 710 seconds followed by a test agent at 910 seconds. Inset expands the oleate testing period to show effects of glutamate (G) and malate (M) individually. Other abbreviations are: NO – no oleate, dBSA – delipidated albumin. C. Group averages for studies such as those in panel B comparing restoration of ΔΨm. Delipidated albumin (dBSA) fully restored ΔΨm in every case. Effects of other agents were calculated relative to the level reached with dBSA. Values are means±SEM for N = 3–5. *P≤0.05, significantly different change of ΔΨm relative to the no test addition starting point. Other abbreviations are: G – glutamate A- α-ketoglutarate, M – malate, AM - α-ketoglutarate+malate, GM – glutamate, ASP – aspartate. D. Effect of malate (M) addition on succinate-supported energization in the absence of exogenous NEFA. Left panel shows tracings illustrating concentration dependence of the malate effects. Numbers adjacent to each tracing are the concentrations of malate added in mM. Right panel shows effect of dBSA.

It has been suggested that NEFA-induced deenergization results from cycling of NEFA across the inner mitochondrial membrane that is at least in part mediated by movement of the NEFA on some of the normal inner membrane anion carriers including the glutamate:aspartate carrier [26], [27] and we previously found that glutamate added to succinate ameliorates deenergization produced both by oleate in normoxic tubules and by H/R [15]. The lesser sensitivity of tubules to oleate in the presence of AMG as compared to succinate seen in the Fig. 1 results would be consistent with this mechanism due to the inclusion of glutamate in the combination. Fig. 2 summarizes studies to further investigate the substrate interactions under conditions that tested the reversibility of oleate-induced deenergization. Based on dose response experiments such as those shown in Fig. 2A, 1 µM oleate was added to tubules energized by succinate after safranin O uptake was complete, followed after an additional 200 seconds by test agents (Fig. 2B). Delipidated albumin (dBSA) as the test agent completely reversed deenergization, while glutamate had a partial effect (Figs. 2B and C). However, aspartate, which should have worked similarly to glutamate if glutamate was acting to oppose NEFA movements via the glutamate:aspartate carrier [15], [26], [27], had no effect to relieve deenergization, nor did αKG. Notably, malate alone and in combination with αKG worsened deenergization, and decreased, but did not eliminate the benefit provided by glutamate. In view of the deleterious effect of malate in the presence of oleate, we then tested adding malate without prior exogenous oleate. The malate similarly deenergized and this deenergization was blocked by binding of endogenous NEFA with dBSA (Fig. 2D). Thus, NEFA were required for the deenergizing effect of malate.

**Figure 3:**
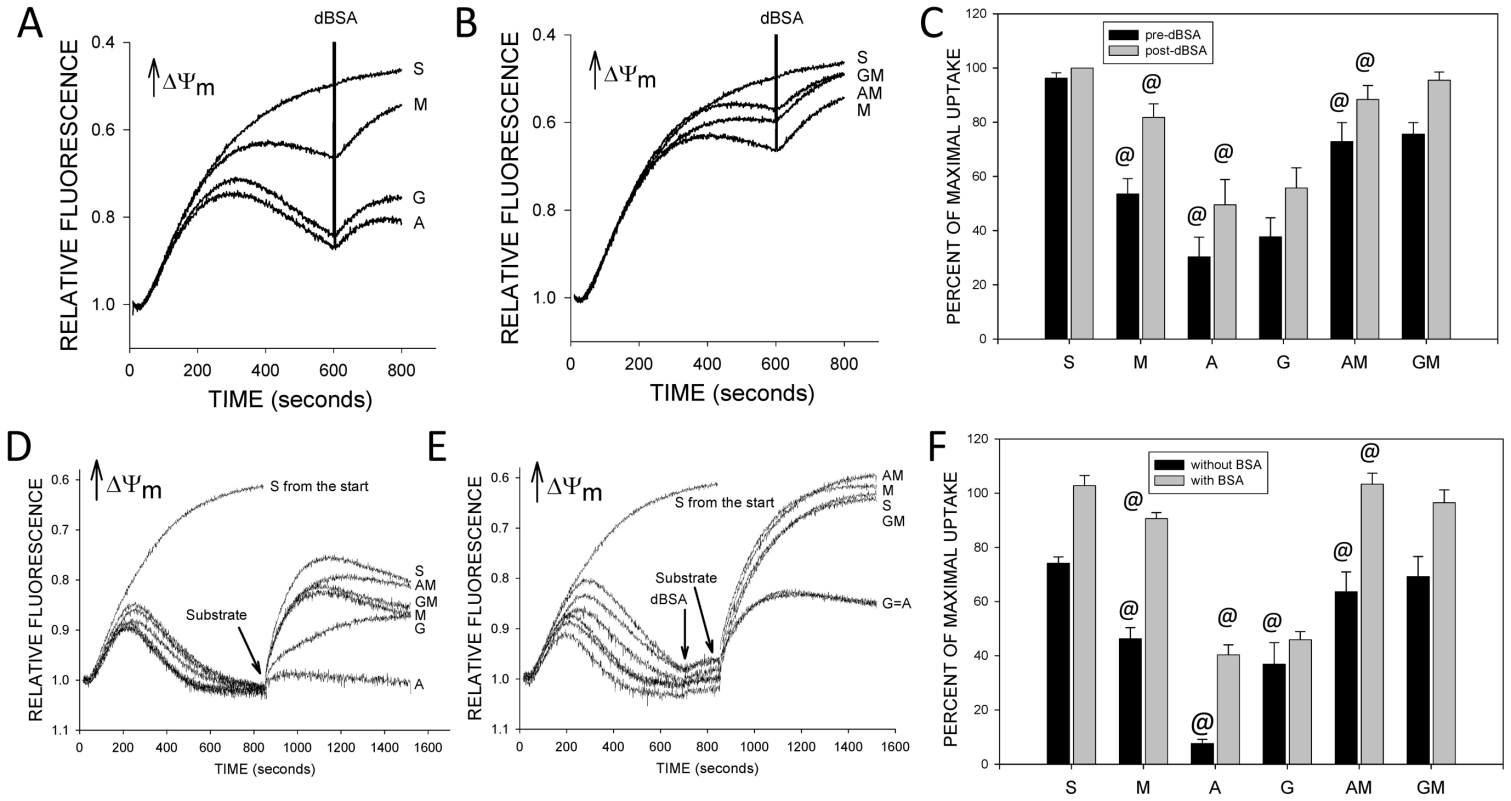
Comparison of substrate support for energization in the absence of exogenous NEFA. Safranin O uptake by permeabilized rabbit tubules was followed in the presence of the indicated substrates. Panels A. B, D, and E are typical tracings. Panels C and F summarize the group results. In the panel A–C studies, the indicated substrates were present from the start and delipidated albumin (dBSA) was added at 600 seconds. In the panel D–F studies endogenous substrates were first depleted by allowing energization in the absence of exogenous substrate, then substrates were added as indicated either without (panel D) or with (panel E) prior dBSA. Abbreviations are: S-succinate, M-malate, A- α-ketoglutarate, G-glutamate, AM-α-ketoglutarate+malate, GM-glutamate+malate. Values in panels C and F are means±SEM for N = 3–5. @ P<0.05 vs. the corresponding group in the preceding set.

Given the surprising results with malate it became relevant to further address the efficacy of a range of substrates to support energization in the system. Fig. 3 summarizes studies of the ability of substrates individually and in combination to primarily support energization. In the Fig. 3A–C studies the test substrates were present from the start. In the figure 3D–F studies endogenous substrates were initially depleted followed by addition of the test substrates. In each type of study, experiments were done with and without dBSA to assess the contribution of endogenous NEFA to the observed behavior. Under all of the conditions studied in Fig. 3 succinate provided the strongest support for energization. When tested as a sole substrate, malate was weaker than succinate but supported energization better than glutamate and αKG. Moreover, unlike its effects to antagonize energization when added to succinate, malate increased energization when added to glutamate and αKG. Under most of the conditions, binding of endogenous NEFA with dBSA improved energization without changing the behavior of the substrates relative to each other. Additional considerations relating to these experiments are addressed below.

### Role of oxaloacetate in the aggravation by malate of NEFA-induced deenergization

**Figure 4:**
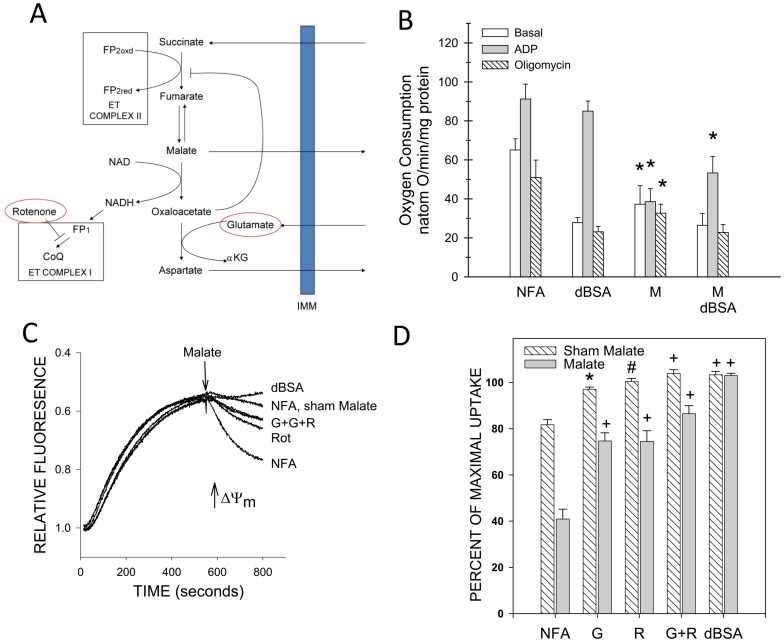
Mediation of malate-induced deenergization by oxaloacetate. A. Pathways by which glutamate and rotenone decrease mitochondrial matrix oxaloacetate accumulation. IMM – inner mitochondrial membrane, ET – electron transport, FP – flavoprotein, αKG – α-ketoglutarate. B. Effects of malate on succinate-supported respiration of rabbit tubules. Basal, ADP-stimulated, and oligomycin-suppressed respiratory rates were measured under the indicated conditions. Abbreviations are NFA – no further additions, dBSA – delipidated bovine serum albumin, M malate (4 mM), M dBSA – malate (4 mM) plus dBSA. Values are means±SEM for N = 3–5. *P<0.05, significantly different from the corresponding addition without malate. C. Representative safranin O uptake tracings showing effects of adding malate to rabbit tubules energized with succinate. Labels indicating the conditions for each experiment are in the order of the tracings from top to bottom. Malate was added at 600 seconds. Abbreviations are dBSA – delipidated bovine serum albumin, M – malate (4 mM), G – glutamate (4 mM), R – rotenone (1 µM), NFA – no further additions. D. Group results for the studies illustrated in panel C. Tubules received either malate or a sham addition at 600 seconds with measurements of the signal at 800 seconds. Data for each condition are presented relative to behavior of the corresponding dBSA sample with sham addition, where no loss of signal occurred. Values are means±SEM on studies from 5 separate preparations. *P<0.05, #P<0.01, or +P<0.001 relative to corresponding NFA group.

The deenergizing effects of malate when combined with succinate could result from inhibition of succinate dehydrogenase by oxaloacetate produced from malate ([28]–[33], Fig. 4A), which would limit succinate-supported respiration and, thereby, support for energization. The effects of added malate in the Fig. 2 studies, thus, would have been due to malate-induced inhibition of succinate-supported respiration that opposes deenergization produced by both exogenous NEFA and by background endogenous NEFA. In support of this mechanism, measurements of respiration showed that a substantial fraction of respiration was being driven by endogenous NEFA since dBSA lowered both basal and oligomycin rates by over 50% (Fig. 4B). Malate inhibited both basal and oligomycin rates when endogenous NEFA were not removed by dBSA, and inhibited ADP-stimulated rates irrespective of dBSA, indicating that it caps maximal respiration independent of the driving force for it. Oxaloacetate accumulation and its inhibitory effect on succinate dehydrogenase can be relieved by use of glutamate to transaminate the oxaloacetate to aspartate, which can then exit the mitochondrial matrix via the glutamate:aspartate carrier, or generation of oxaloacetate can be prevented by rotenone (Fig. 4A). As shown in Figs. 4C and 4D, both rotenone and glutamate similarly ameliorated deenergization induced by malate in tubules respiring on succinate and they were not additive. Unlike dBSA, however, they did not completely prevent malate-induced deenergization. Glutamate, rotenone and dBSA also significantly ameliorated the milder, NEFA-dependent spontaneous decay of energization that occurred in the absence of added malate (Fig. 4D).

### Behavior of mouse tubules

**Figure 5:**
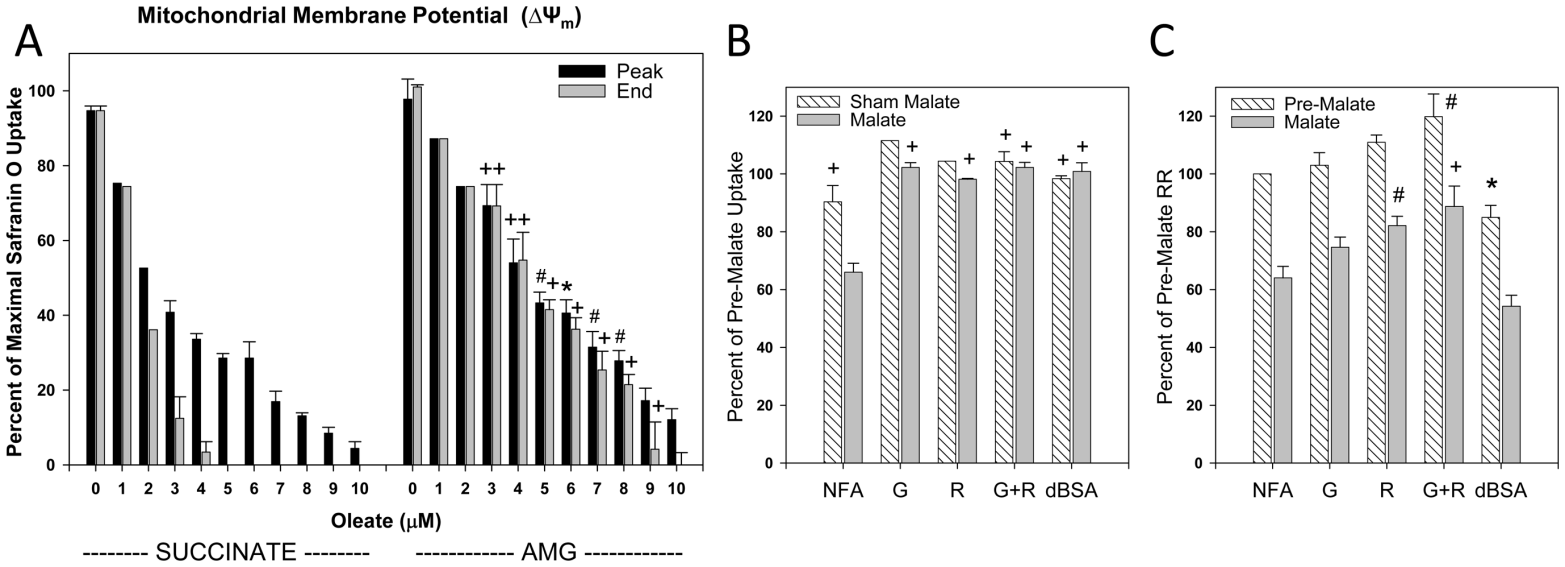
Effects of oleate and malate on energization of mouse tubules. A. Concentration dependence of effects of oleate on energization of permeabilized mouse tubules measured with safranin O uptake supported by either succinate or the combination of complex I dependent substrates, α-ketoglutarate, malate, and glutamate. “Peak” indicates the maximal uptake compared to the uptake seen without added oleate using succinate as substrate in the presence of delipidated albumin to eliminate the effect of endogenous fatty acids. “End” indicates the final level reached at the end of the 600 second measurement period, which can be less than the peak if there has been decay of ΔΨm. Values are means±SEM for N = 3. *P<0.05, #P<0.01, +P<0.001 vs. corresponding succinate group. B and C. Effects of malate on succinate-supported energization measured using safranin O uptake (Panel B) and respiration (Panel C). Permeabilized tubules were incubated with succinate and the indicated test agents for 350 seconds (Pre-Malate period) followed by addition of either sham malate or malate for 200 seconds with measurement of safranin uptake and respiratory rate (RR) at the end of that period. Values are compared to those measured for the no further addition (NFA) group at the end of the ‘Pre-Malate’ period. Other abbreviations are: G – glutamate, R - rotenone, G+R =  glutamate+rotenone, dBSA – delipidated bovine serum albumin. For the safranin O uptakes, values are means±SEM for N = 2–3 for sham malate and 3–5 for malate. +P<0.001 for conditions with N≥3 vs. the corresponding NFA malate condition. For the respiratory rates, values are means±SEM for N = 4, *P<0.05, #P<0.01, +P<0.001 vs. corresponding NFA group.

Similar to the rabbit tubules, mouse tubules displayed less sensitivity to oleate-induced deenergization in the presence of AMG as substrates as compared to succinate (Fig. 5A). Also similar to the rabbit tubules, adding malate to mouse tubules respiring on succinate deenergized them (Fig. 5B) and slowed respiration (Fig. 5C). Glutamate, rotenone and dBSA all completely prevented the deenergizing effect of late malate addition to the mouse tubules and the spontaneous decay that occurred in the absence of added malate (Fig. 5B). Glutamate and rotenone also stimulated respiration in the malate-treated mouse tubules but did not eliminate the difference from tubules without malate (Fig. 5C).

### Basis for amelioration of NEFA-induced deenergization by glutamate

**Figure 6:**
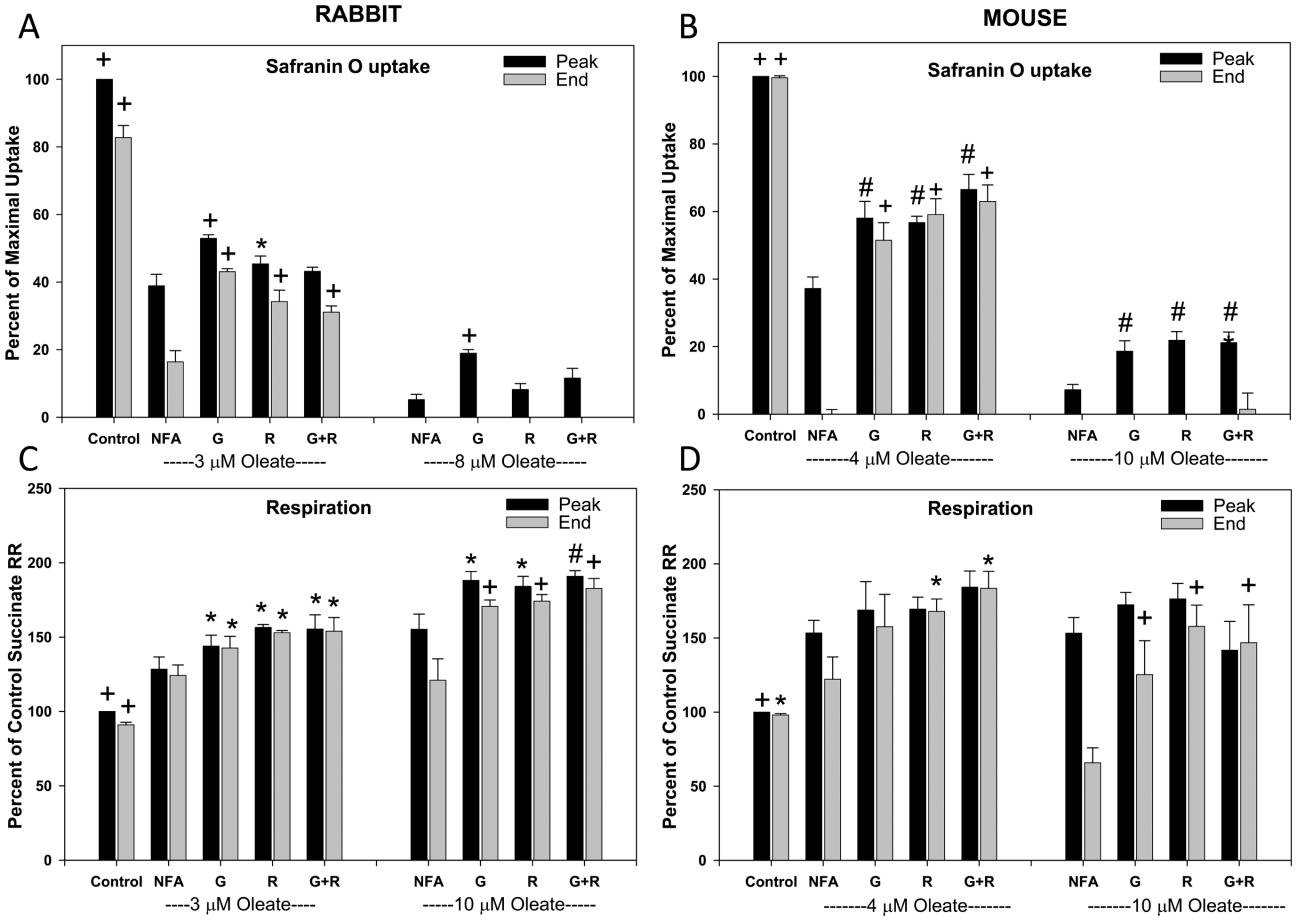
Effect of glutamate and rotenone on energization and respiration supported by succinate in rabbit and mouse tubules. Safranin O uptakes and respiratory rates (RR) of permeabilized tubules were measured either under control conditions or in the presence of the indicated concentrations of oleate with either no further additions (NFA) or glutamate (G), rotenone (R) or glutamate+rotenone (G+R). “Peak” indicates the maximal uptake or RR during the measurement period. “End” indicates the uptake level or RR reached at the end of the second measurement period, which is less than the peak for conditions where there has been decay of ΔΨm or RR. Concentrations of oleate were chosen to give moderate deenergization (3 µM for rabbit, 4 µM for mouse) or severe deenergization (8 µM for rabbit, 10 µM for mouse). The high oleate concentration used for the rabbit RR studies was increased to 10 µM to provide more consistent decreases of the end RR to allow assessment of agents that ameliorate it. Values are means±SEM for N = 3 for both types of rabbit studies, 5–7 for the mouse safranin O uptakes and 4–5 for the mouse respiratory rates. *P<0.05, #P<0.01, +P<0.001 vs. corresponding NFA group.

The observations in Fig. 4 raise the possibility that the benefit of glutamate for NEFA-induced deenergization in the presence of succinate (Fig. 2C and [15]) is mediated not by prevention of NEFA cycling but instead by relieving respiratory inhibition. The two mechanisms predict opposite effects of glutamate on succinate-supported respiration in the presence of NEFA. If glutamate relieves respiratory inhibition it should enhance or at least maintain stimulation of respiration by NEFA; if it opposes NEFA cycling across the membrane it should block respiratory stimulation by NEFA. The Fig. 6 studies assess the effects of glutamate on oleate-induced deenergization and respiratory stimulation and compare them with those of rotenone. Permeabilized rabbit and mouse tubules were treated with low and high concentrations of oleate chosen to optimize effects in each type of tubule to either partially deenergize (3 or 4 µM) or completely deenergize (8–10 µM) and either no further addition of test agents or glutamate alone, rotenone alone or glutamate+rotenone. Each of the agents improved energization at both the low and high concentrations of oleate (Figs. 6A and 6B). At the low concentrations of oleate glutamate did not significantly change respiration in either type of tubule. At 10 µM oleate, both glutamate and rotenone similarly increased respiration in both the rabbit and mouse tubules and were not additive. Overall, the data from these studies with succinate and oleate show that glutamate does not inhibit respiration as would be expected if it were blocking NEFA cycling. Instead, respiration with glutamate is either maintained or increased. Rotenone stimulates respiration as expected if it acts by relieving inhibition by malate.

**Figure 7:**
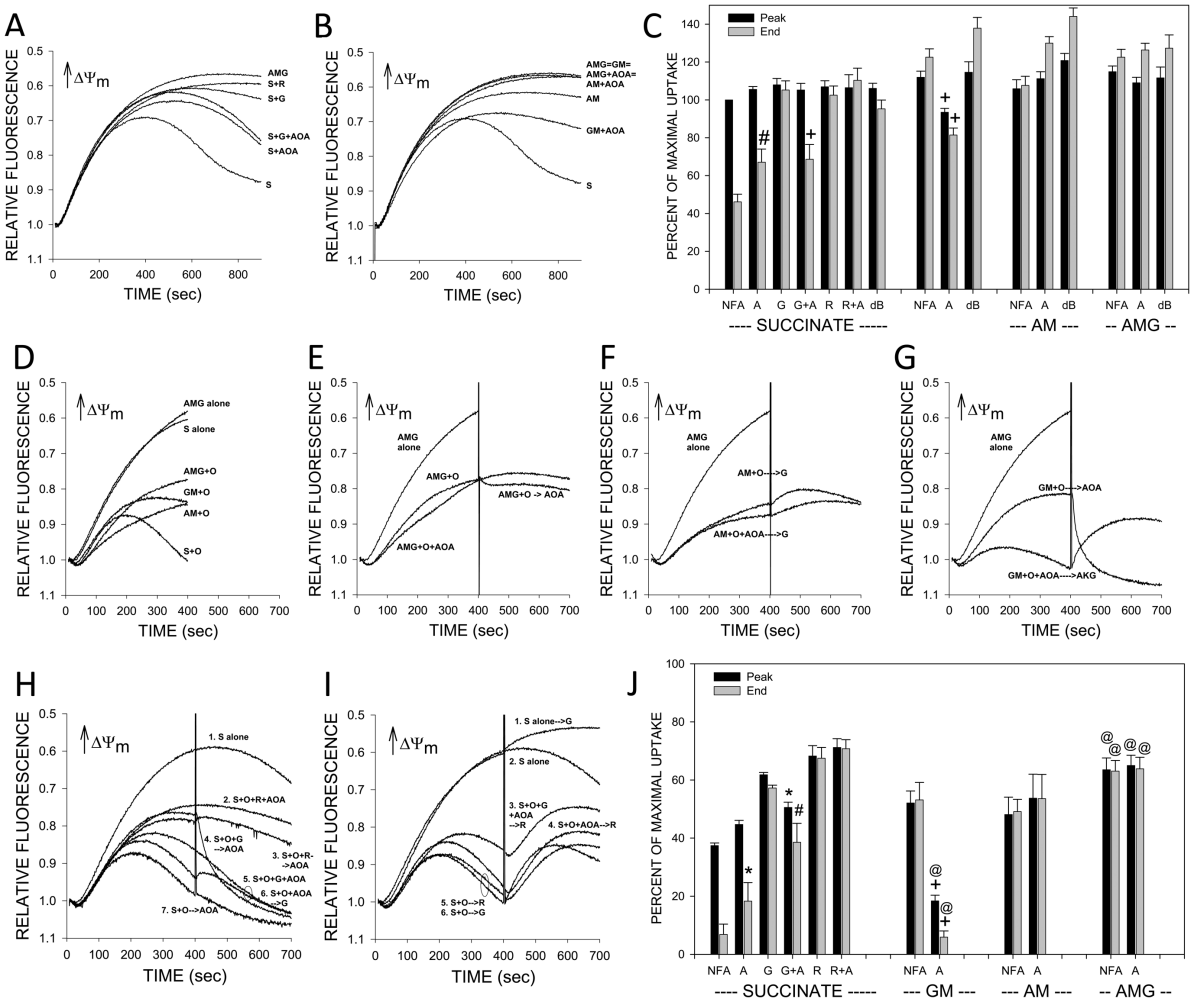
Effects of transaminase inhibition by aminooxyacetate on energization of mouse tubules with and without oleate addition. Panels A–C. Studies of extended incubation without exogenous fatty acids (900 seconds). Panels A and B show representative tracings. Panel C summarizes group averages compared to the maximal uptake during the period for succinate alone. ‘Peak’ indicates maximal uptake for each condition. ‘End’ indicates uptake at 900 seconds. Agents tested and abbreviations for them are: S - succinate, M - malate, A - α-ketoglutarate, G - glutamate, AM - α-ketoglutarate+malate, GM - glutamate+malate, AMG - α-ketoglutarate+malate+glutamate, A or AOA – aminooxyacetate (4 mM), O – oleate (4 µM), R – rotenone, dB – delipidated bovine serum albumin. For the group data in panel C, values are means±SEM for N = 3–7, #P<0.01, +P<0.001 vs. corresponding group without aminooxyacetate. Panels D–J. Studies testing oleate. Panels D–I are representative tracings. Panel J summarizes group averages for behavior during the first 400 seconds in a format identical to that used for the corresponding studies without oleate in Panel C. All studies were 700 seconds in duration with a second experimental condition indicated in the panel introduced at 400 seconds. Panel D compares energization during the first 400 seconds with and without oleate for each test substrate. Panels E–G show effects of adding aminooxyacetate on the energization supported by complex I substrates. Panels H and I show effects of aminooxyacetate on succinate-supported energization with and without glutamate and rotenone. Agents tested and abbreviations used are as described for Panels A–C with additional use of O – oleate (4 µM). For the group data in panel J values are means±SEM for N = 3, *P<0.05, #P<0.01, +P<0.001 vs. corresponding group without AOA; @p<0.05 vs. corresponding ‘AM’ group.

The mechanism for the glutamate effects is further assessed in the Fig. 7 studies using mouse tubules to test the transaminase inhibitor aminooxyacetate (AOA), which should block glutamate effects that are mediated by transamination of oxaloacetate (Fig. 4A). Panels A–C summarize the results of studies without addition of oleate. These studies were continued beyond the point of maximal safranin O uptake (400 seconds) for a total of 700 seconds to allow testing of additional maneuvers. Tubules supported by succinate spontaneously deenergized even without added oleate over this period (Fig. 7A). The deenergization was prevented by either glutamate, rotenone, or dBSA (Figs. 7A and 7C) implying involvement of endogenous fatty acids and modulation by oxaloacetate accumulation. AOA itself slightly ameliorated the spontaneous deenergization with succinate, but also fully blocked the benefit of glutamate (Figs. 7A and 7C). It did not alter the effects of rotenone. All of the complex I dependent substrate combinations, glutamate+malate (GM), αKG+malate (AM), and αKG+malate+glutamate (AMG), showed stronger energization than succinate that did not decay (Figs. 7B and 7C). Differences between them were not significant. AOA decreased energization with GM, consistent with a requirement for transamination to allow full utilization of the glutamate, but not with AM or AMG (Figs. 7B and 7C).

Studies testing the effect of AOA on the response to oleate are summarized in Figs. 7D–7J. Similar to the previous studies in Figs 5 and 6 testing oleate addition, oleate deenergized more strongly with succinate than with AMG (Figs. 7D and 7J). Oleate tended to deenergize slightly more with AM than with GM or AMG, but the difference was significant only for AMG (Figs. 7D and 7J). As in the previous Fig. 6 study using succinate and oleate, with succinate as substrate both glutamate and rotenone ameliorated oleate-induced deenergization to a similar degree when present from the start of the experiment and also, as newly shown here, when added late (Figs. 7H, 7I, and 7J). AOA decreased and strongly reversed the effect of glutamate, but did not alter the effect of rotenone. AOA itself mildly ameliorated oleate induced deenergization when present from the start of the experiment. Figs. 7E–G summarize parallel studies with the complex I substrates plus oleate. AOA limited, but did not entirely prevent the benefit of adding glutamate to AM (Figs. 7E, 7F and 7J). It strongly impaired energization supported by GM in the presence of oleate (7G and 7J). Thus, prevention of transamination of oxaloacetate by glutamate using AOA limits the benefit of glutamate in all settings. Although AOA can have off target actions not mediated by inhibition of transamination [34], they are unlikely to have contributed to the acute effects under these experimental conditions and the conclusions from these AOA studies are consistent with these reached independently using the other approaches.

### Studies of hypoxia/reoxygenation

**Figure 8:**
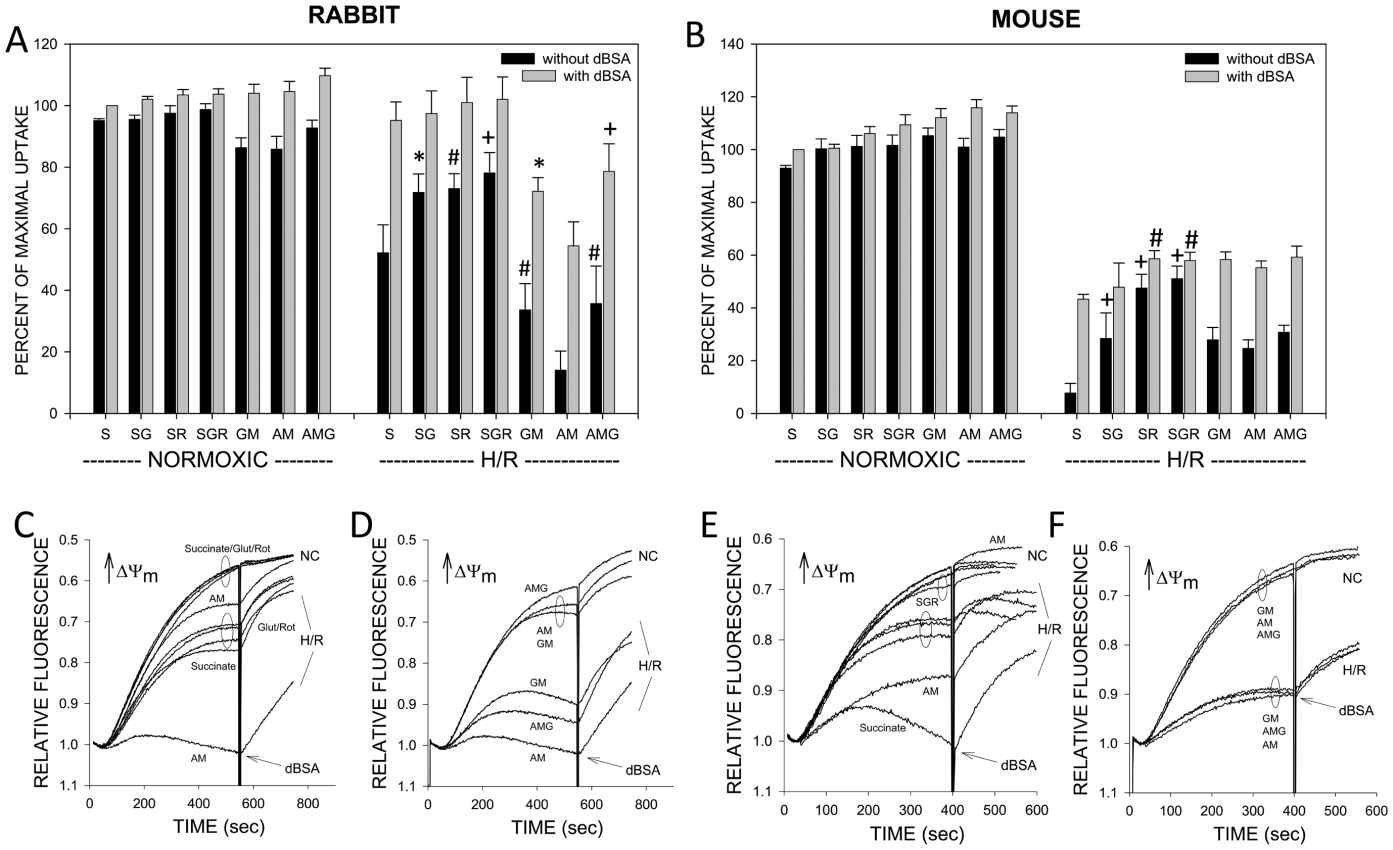
Support of energization by succinate compared to complex I substrates after H/R in rabbit and mouse tubules. Tubules were subjected to either normoxic incubation or to 67.5(Rabbit, Panels A, C, and D) or 30 min. hypoxia (Mouse, panels, B, E, and F) followed by 60 min reoxygenation then measurement of energization using safranin O uptake with either succinate (S) glutamate+malate (GM), α-ketoglutarate+malate (AM) or α-ketoglutarate+malate+glutamate. Succinate was also studied with addition of either glutamate (SG), rotenone (1 µM, SR) or both glutamate and rotenone (SGR). Measurements were made initially without delipidated albumin (dBSA) followed by its addition (0.5 mg/ml) at the vertical marks in the tracings. Group averages±SEM for N = 5 for both rabbit and mouse are summarized in panels A and B. Typical tracings are shown in panels C–F. Statistics shown in panels A and B indicate values significantly different from the corresponding S group for the succinate studies or AM group for the complex I substrate studies at either *P<0.05, #P<0.01, or +P<0.001. Other statistical analysis indicated that all H/R conditions were significantly different (P<0.01) from the corresponding normoxic conditions except for the rabbit SG, SR and SGR groups with dBSA, which completely recovered. dBSA significantly increased energization (P<0.01) in all rabbit studies except for the normoxic succinate groups. In the mouse tubules, dBSA significantly improved energization of normoxic tubules with AM (P<0.05) and in all hypoxic groups (P<0.01) except for SR and SGR. In both normoxic and hypoxic rabbit tubules, energization with complex I substrates was poorer than with succinate (P<0.05) except for normoxic tubules in the presence of dBSA. In normoxic mouse tubules, complex I supported energization did not differ from succinate, but after H/R complex I rates energization was better than with succinate alone irrespective of the presence of dBSA (P<0.05). Relative to the other succinate conditions, complex I energization was either weaker (P<0.01 relative to SR and SGR without dBSA) or unchanged.

Release of NEFA induces sustained reversible deenergization during reoxygenation of both rabbit and mouse tubules [9], [11], [15]. The deenergization measured with safranin O in the permeabilized tubules is closely associated with changes of JC-1 fluorescence in unpermeabilized tubules and ATP levels of intact tubules, which are in turn highly predictive of recovery from hypoxia-induced effects on ultrastructure, tyrosine phosphorylation and focal adhesions, and ability to remain viable [1]–[3], [8]–[11], [15], [19], [23]–[25]. Although all of these other parameters were not measured in the current studies, ATP levels and JC-1 fluorescence were monitored in every experiment and displayed the behavior expected from the extensive prior work with the system (not shown). The effects of H/R on energization measured with safranin O uptake in the present studies are illustrated and further investigated in the work summarized in Fig. 8. For these experiments, tubules sampled at the end of H/R were permeabilized and followed to the point of maximal safranin O uptake then had dBSA added to assess the effect of removing NEFA as illustrated in the representative tracings shown in panels C–F. Experimental conditions assessed effects of glutamate and rotenone during succinate-supported energization and several combinations of complex I-dependent substrates based on the results of the Fig. 5–7 studies showing uniquely informative aspects of behavior under each of the conditions.

Normoxic tubules from both species were well energized under all substrate conditions tested and energization supported by succinate was not modified by glutamate or rotenone. dBSA had a small but statistically significant effect to improve energization over the time frames of these studies only in normoxic rabbit tubules supported by complex I substrates. H/R resulted in deenergization that was reversed by dBSA with nearly complete restoration of energization by dBSA in the rabbit tubules, but only partial restoration in the mouse tubules, consistent with our prior work implicating NEFA as major factors in the deenergization [9], [11], [15]. Both glutamate and rotenone significantly improved energization in tubules respiring on succinate after H/R with relatively greater effects in the mouse tubules. In the rabbit tubules, complex I substrates gave weaker energization than all the succinate conditions after H/R. GM and AMG in the rabbit tubules were substantially stronger than AM. dBSA significantly improved energization under all conditions in the rabbit tubules. In the mouse tubules, complex-I dependent substrates gave better energization than succinate alone after H/R, but were not as good as succinate combined with glutamate and rotenone. AMG and GM tended to be better than AM, but the differences were small. dBSA significantly improved energization under all substrate conditions except the groups with succinate+rotenone. Taken together, these data indicate that the same substrate interactions and inhibitory effects of oxaloacetate on energization seen in oleate-treated normoxic tubules were present after H/R-induced increases of endogenous NEFA.

**Figure 9:**
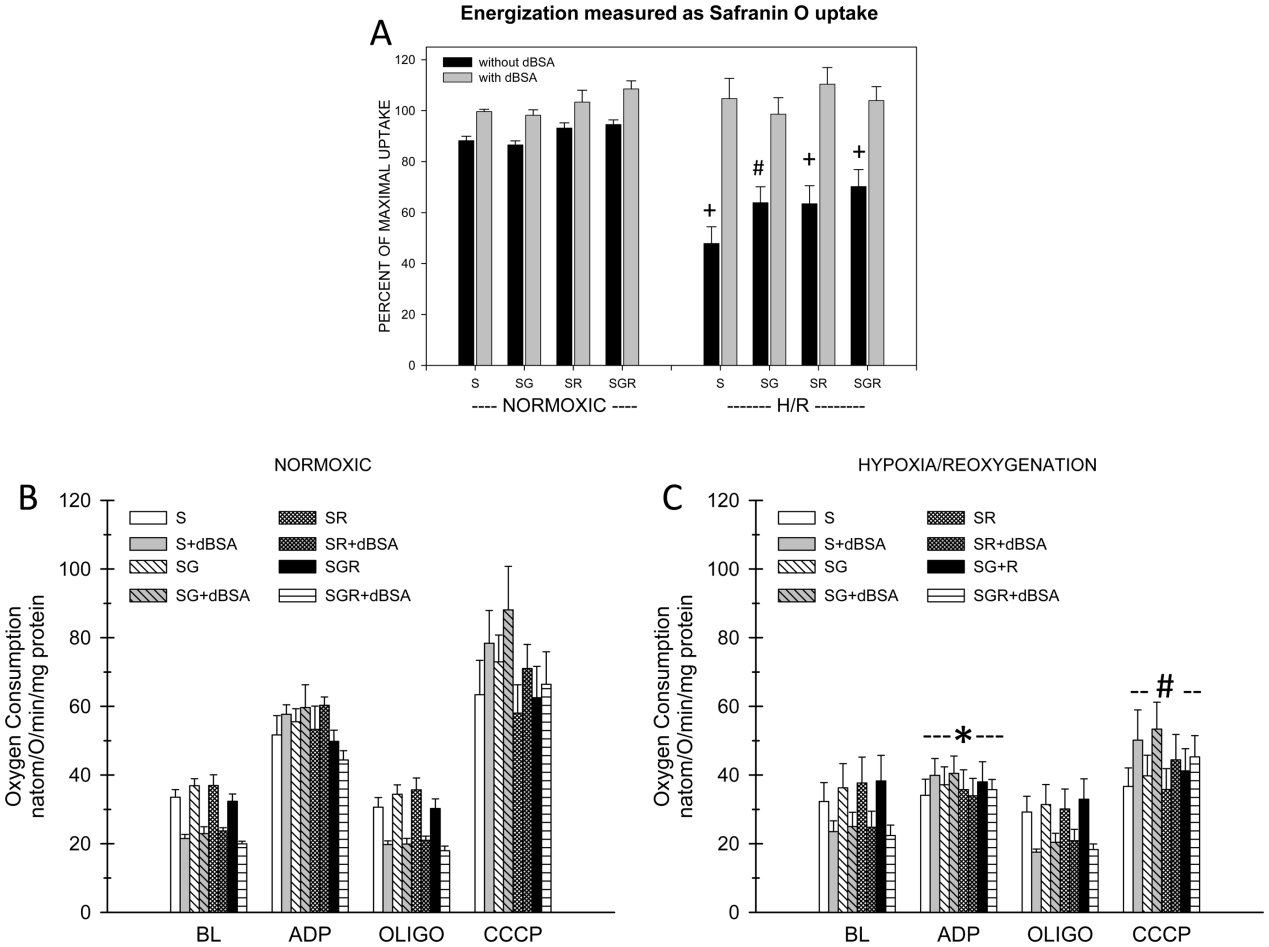
Effects of glutamate and rotenone on succinate-supported respiration of normoxic and H/R rabbit tubules. A. Measurements of energization using safranin O uptake done in parallel with the respiration studies on the same preparations. Abbreviations for the experimental groups testing different conditions during safranin O uptake are as for Fig. 8. Tubules were subjected to 67.5±SEM for N = 4. Figure symbols indicate #P<0.01 or +P<0.001 vs. corresponding normoxic values. Values for glutamate and rotenone-treated H/R groups without dBSA were significantly different from the corresponding group with succinate alone, P<0.05. Delipidated albumin (dBSA) significantly improved energization under all the H/R conditions (P<0.001). B. Measurements of respiration. Oxygen consumption was assessed sequentially under basal conditions, then after addition of ADP to stimulate oxidative phosphorylation, then during suppression of the ADP-induced oxidative phosphorylation by oligomycin (OLIGO), then during maximally-stimulated uncoupled respiration produced by carbonyl cyanide-m-chlorophenylhydrazone (CCCP). Studies were done with and without addition of dBSA on tubules previously subjected to either normoxic incubation or H/R. Values are means±SEM for N = 4. There were no significant effects of glutamate and/or rotenone under any condition except for a small decrease of the ADP rate in the normoxic glutamate+rotenone+dBSA group (P<0.05). Basal and oligomycin rates did not significantly differ between normoxic and H/R tubules. dBSA significantly lowered the basal and oligomycin rates in all groups (P<0.001 normoxic, P<0.01 hypoxic). ADP and CCCP rates of H/R tubules were significantly lower than the corresponding normoxic at *P<0.05 or #P<0.01 under all conditions. dBSA did not significantly affect the ADP rates, but it significantly increased the CCCP rates in the normoxic S and SR groups and the H/R S, SG, and SR groups, Ps<0.05 or better.

**Figure 10:**
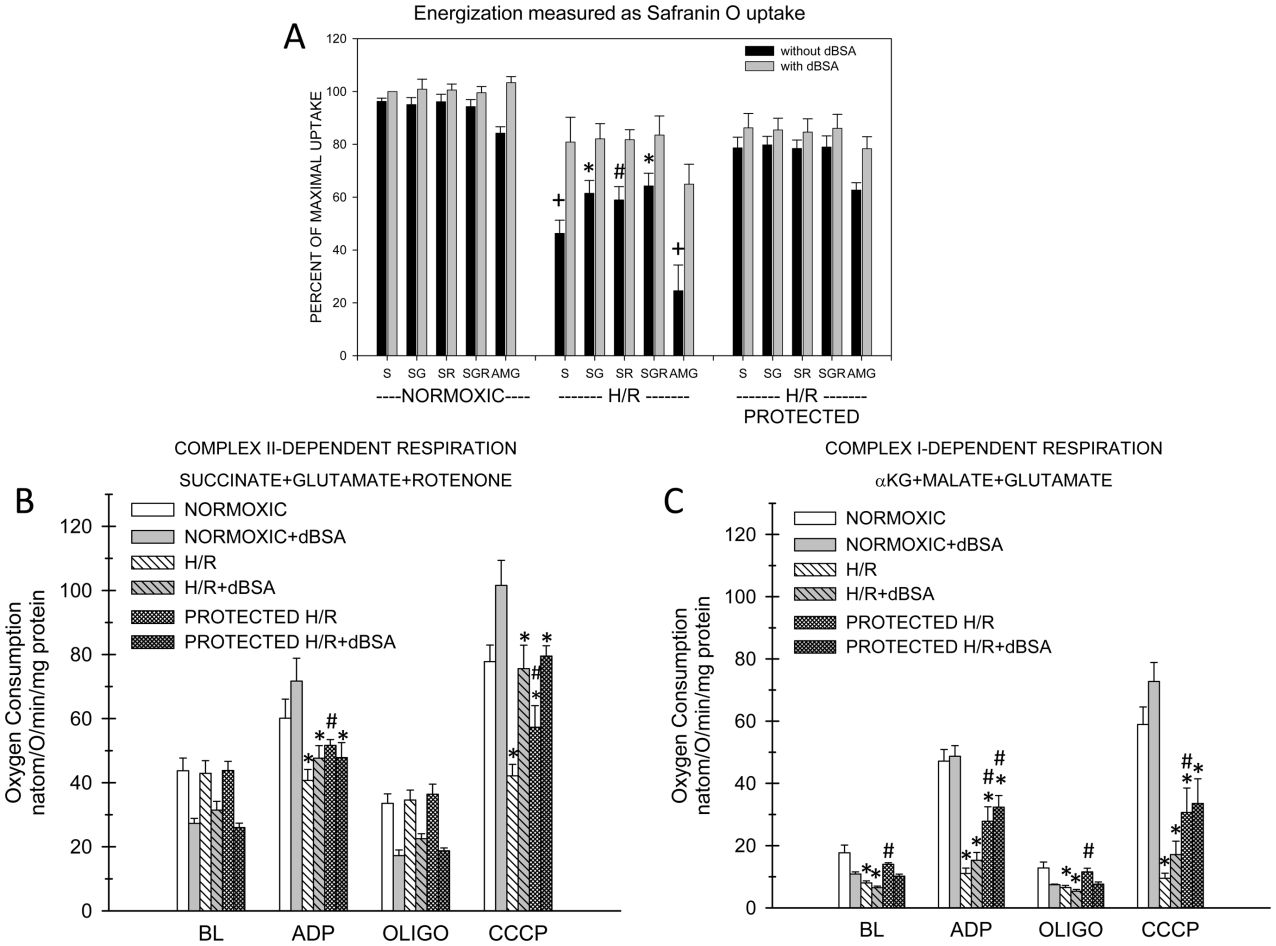
Energization and respiration after H/R of unprotected and substrate-protected rabbit tubules supported by succinate or complex I-dependent substrates. These experiments were done similarly to those in Fig. 9, except they compared the behavior of complex II-dependent respiration with complex I and also tested tubules that were protected by dBSA+αKG/MAL in the incubation flasks during the 60 min. reoxygenation period. A. Measurements of energization using safranin O uptake. Abbreviations for the experimental groups testing different conditions during safranin O uptake are as for Fig. 8. Values are means±SEM for N = 4. All H/R groups except protected tubules with succinate+BSA were significantly different from the corresponding normoxic groups at P<0.001 for unprotected flasks without dBSA and P<0.05 for all other groups. Statistical symbols shown in the figure indicate: *P<0.05, #P<0.01, +P<0.001 vs. corresponding protected flask. dBSA significantly increased energization in all AMG groups and in succinate groups from unprotected tubules, P<0.01. SG, SR, and SGR without dBSA had significantly better uptake than S alone in the unprotected tubules (P<0.05), and AMG without dBSA supported energization after H/R less strongly than S alone in both protected and unprotected tubules, P<0.01. B. Measurements of respiration. Oxygen consumption was measured with either succinate+glutamate+rotenone (SGR) or α-ketoglutarate+malate+glutamate (AMG) following the same experimental sequence as described for Fig. 9. Values are means±SEM for N = 4. SGR rates were significantly greater than the corresponding AMG rates under all conditions, P<0.01. Statistical symbols shown in the figure indicate: *significantly different from normoxic at P<0.01 (SGR studies) or P<0.05 (AMG), #significantly different from corresponding unprotected group at P<0.01 (SGR studies) or P<0.05 (AMG studies). All basal and oligomycin rates with dBSA were significantly lower than the corresponding rates without dBSA (P<0.02) except for the oligomycin rate of the AMG H/R group. dBSA significantly increased the succinate-supported CCCP rates for all groups and the AMG-supported CCCP rate of the normoxic tubules.

Figs. 9 and 10 summarize measurements of succinate-supported respiration in rabbit tubules after control normoxic incubation or H/R along with the changes of energization measured in those experiments. Reoxygenation was assessed both without and with protective agents (dBSA+αKG/MAL) in the flasks to promote recovery. Respiration was measured both without and with dBSA under basal conditions, then during ADP induced stimulation (corresponding to State 3 of isolated mitochondria), then after suppression of ADP stimulation using the F_1_F_O_-ATPase inhibitor, oligomycin (corresponding to State 4 of isolated mitochondria), then after re-stimulation with the protonophoric uncoupler CCCP.

Fig. 9 shows results for studies with rabbit tubules in which succinate-supported respiration was assessed with glutamate and rotenone separately and in combination after control normoxic incubation as compared to unprotected recovery after H/R. Fig. 10 shows the results of separate experiments in which respiration in the presence of succinate+glutamate+rotenone was compared to the complex I dependent combination, AMG, and was assessed after both unprotected and protected recovery. Figs. 9A and 10A additionally show the results of energization measurements that were done on the same samples as the respiration measurements and indicate high consistency of expression of effects relative to the separate energization study in Fig. 8.

After normoxic incubation of rabbit tubules, ADP and CCCP stimulated respiration as expected for induction of oxidative phosphorylation by ADP and uncoupling by CCCP (Fig. 9B). The stimulation by ADP was fully suppressed by oligomycin. dBSA decreased basal and oligomycin rates by 30%, consistent with the presence of background uncoupling by endogenous NEFA. dBSA did not significantly affect the ADP rates, but significantly increased the CCCP rates with succinate alone and succinate+rotenone. None of the rates were consistently or significantly affected by glutamate or rotenone individually or in combination.

After H/R, basal and oligomycin rates of the rabbit tubules supported by succinate were similar to the control normoxic rates, but ADP and CCCP rates were only minimally further increased relative to basal and reached only about 50% of the rates of normoxic tubules in the Fig. 9C studies and 71% in the Fig. 10B studies. Basal and oligomycin rates were decreased by dBSA, but no more than in normoxic controls. dBSA did not significantly affect ADP rates, but moderately increased CCCP rates with succinate alone, succinate+glutamate and succinate+rotenone. Tubules that were protected with dBSA+ αKG/MAL during reoxygenation did not show any differences of succinate-supported rates relative to unprotected tubules. Basal rates trended up with glutamate and rotenone, but the differences did not reach statistical significance.

For the complex I substrates, normoxic rates of the rabbit tubules were significantly less than the corresponding succinate rates (Fig. 10b, basal - 40.1%, ADP - 79.5%, oligomycin 37.4%, CCCP - 75.7%, P<0.001). They were more severely decreased by H/R, particularly the ADP and CCCP rates which dropped to 23% and 16% of the corresponding normoxic rates respectively. Tubules that were protected with dBSA+αKG/MAL during reoxygenation had significant increases of complex I-supported rates with the largest effects on the ADP and CCCP rates.

**Figure 11:**
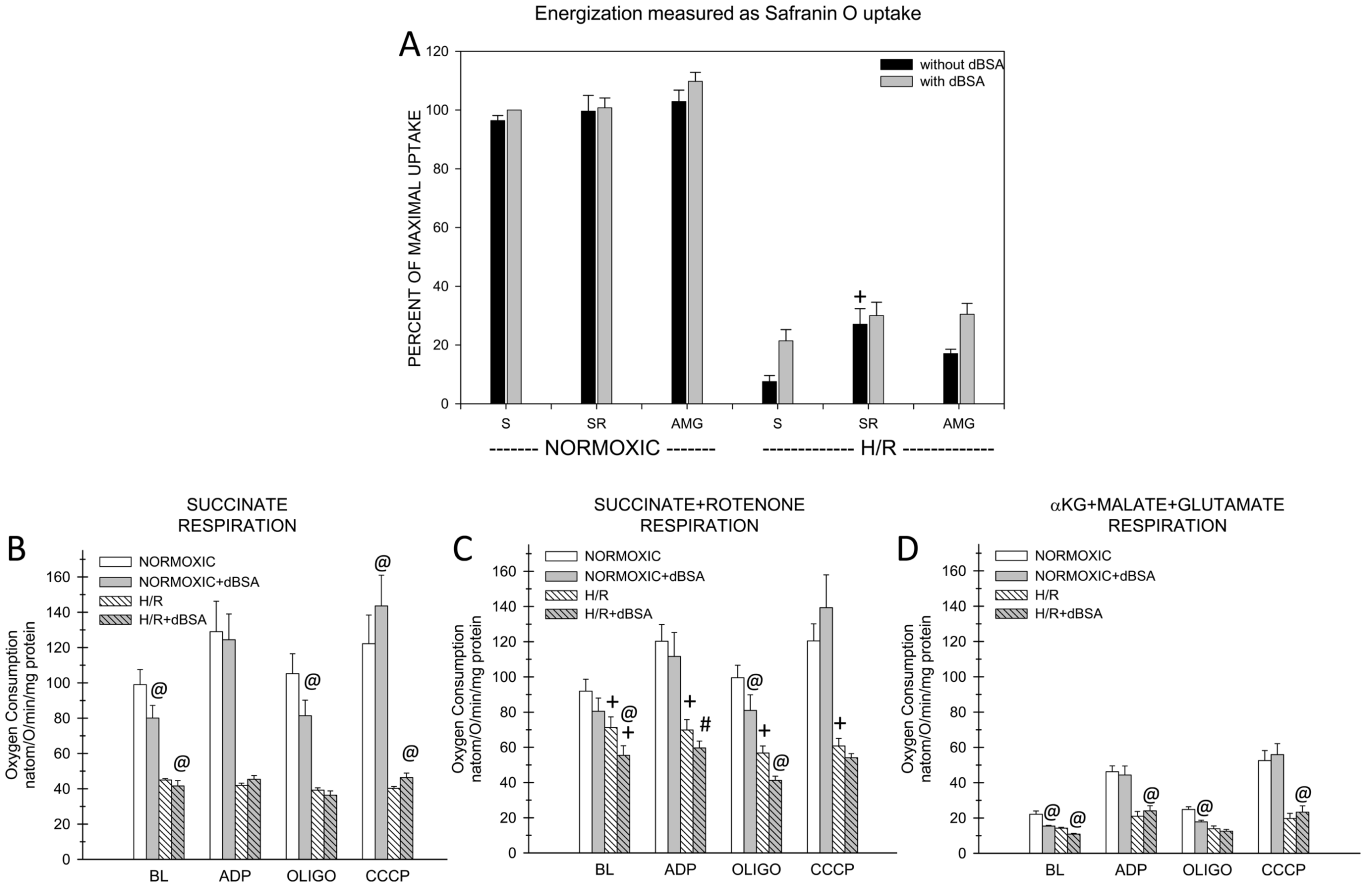
Studies of energization and respiration after H/R of mouse tubules supported by succinate or complex I-dependent substrates. These experiments were done similarly to those in Figs. 9 and 10, except tubules were subjected to 30-supported energization are as for Fig. 8. Values are means±SEM for N = 4. All H/R groups were significantly different from the corresponding normoxic groups at P<0.001. dBSA significantly increased energization only in the succinate alone (S) and AMG groups. Statistical symbols shown in the figure indicate: *P<0.05 or #P<0.01 vs. corresponding ‘S’ group. B. Measurements of respiration. Oxygen consumption was measured with either succinate alone or succinate+rotenone (SR) or α-ketoglutarate+malate+glutamate (AMG) following the same experimental sequence as described for Fig. 10. All H/R rates were significantly different from the corresponding normoxic rates, P<0.001. All succinate alone and SR rates were significantly greater than the corresponding AMG rates at P<0.001. Figure symbols indicate: #P<0.01, +P<0.001, SR group values that were significantly different from corresponding succinate alone values; @, significant effects of dBSA vs. the corresponding condition without it. The dBSA effects for basal rates were P<0.05 for normoxic succinate alone, P<0.001 for normoxic AMG and P<0.01 for all H/R groups. For oligomycin rates, they were P<0.01 for all normoxic groups and the H/R SR study. The CCCP rate with dBSA was different at P<0.05 for the succinate alone group.

Thus, the major characteristics of the respiratory patterns in the rabbit tubules are the absence of modification of succinate respiration by glutamate or rotenone in both normoxic and H/R tubules, inhibition of maximal respiratory rates after H/R that is not modified by removing NEFA acutely with dBSA, and more severe inhibition of complex I-dependent respiration that can be ameliorated by incubating tubules under protected conditions during reoxygenation,

Results for mouse tubule studies testing the effect of H/R are summarized in Fig. 11. Energization was more severely inhibited after H/R than in the Fig 8 mouse studies, but patterns of changes with rotenone, AMG, and dBSA were similar in that energization after H/R was particularly poor with succinate, but this was improved by rotenone (Fig. 11A). dBSA improved energization with succinate alone and AMG, but not with succinate+rotenone.

Under normoxic control incubation conditions, mouse tubules had absolute succinate-supported basal and oligomycin respiratory rates 2.5x greater than rabbit tubules, but similar rates when these respiratory conditions were supported by AMG. ADP and CCCP rates with succinate were also higher for the mouse than for the rabbit but not by the same degree (compare panels B–D of Fig. 11, with panels B and C of Figs. 9 and 10 taking into account the different scales). Binding NEFA with dBSA significantly lowered basal and oligomycin rates under all conditions except for succinate+rotenone (P<0.05 or better), but not to the same degree as in rabbit tubules. As in the rabbit, dBSA tended to increase the CCCP rate, but this was only significant for succinate alone (P<0.05).

Unlike the rabbit tubules, basal and oligomycin rates of the mouse tubules with succinate alone were substantially inhibited after H/R (Fig. 11B). This inhibition was relieved by rotenone (Fig. 11C). As for the rabbit tubules, stimulation of respiration by ADP and CCCP in the presence of succinate was strongly suppressed in the mouse tubules after H/R, although it was somewhat alleviated in the mouse by rotenone. Complex I-dependent rates, particularly the ADP and CCCP rates, were more inhibited (to 45% and 31% of normoxic) than succinate rates (58% and 50% of normoxic), but the complex I H/R effects were proportionately less than for the rabbit. dBSA had smaller effects after H/R than after normoxic incubation. As for normoxic mouse tubules, effects of dBSA on respiration of the mouse tubules after H/R were less than seen for the rabbit. For succinate alone, dBSA slightly inhibited the basal rate (P<0.05) and slightly stimulated the CCCP rate (P<0.05). For succinate+rotenone it inhibited the basal rate (P<0.05). For AMG it inhibited the basal rate (P<0.001) and slightly stimulated the ADP (P<0.05) and CCCP rates (P<0.01).

The major characteristics of the respiratory patterns in the mouse tubules were the higher succinate-supported respiratory rates overall, particularly the basal and oligomycin rates, the sensitivity of these rates to rotenone particularly after H/R, the lesser proportional effect of H/R to inhibit the complex I rates than in the rabbit, and the lack of effect of dBSA to ameliorate respiratory inhibition after H/R.

## Discussion

The main findings are: 1) Generation of oxaloacetate from succinate can substantially limit energization and respiration particularly in the presence of NEFA where increased substrate utilization is required to compensate for uncoupling. Lowering oxaloacetate by transamination rather than competition with NEFA for cycling on anion carriers accounts for most of the benefit for energetics of adding glutamate in the presence of succinate. Mouse tubules have particularly high rates of succinate-supported respiration that are impacted by this behavior. 2) When the inhibitory effects of oxaloacetate accumulation are limited by either transaminating it with glutamate or preventing its generation with rotenone, exogenous NEFA primarily stimulate respiration of healthy permeabilized tubules with both succinate and complex I-dependent substrates at concentrations up to 10 µM that are relevant to the levels reached during ischemia in vivo and hypoxia in vitro [9] and degrees of deenergization are similar with both types of substrates. 3) After H/R, succinate supported energization is more improved by limiting oxaloacetate accumulation than in normoxic tubules, consistent with the increased NEFA present in that setting. This is paralleled by increases of succinate-supported respiration in the mouse, but not in the rabbit. However, basal respiratory rates, which correspond to respiration during measurements of energization with safranin O uptake, are not stimulated by NEFA accumulation after H/R relative to normoxic tubules as they are when normoxic tubules are treated with oleate. ADP and CCCP-stimulated rates are strongly inhibited after H/R with greater effects proportionately for complex I-dependent substrates and this inhibition is not relieved by removing excess NEFA with delipidated albumin. Rates are improved by maneuvers that improve energetic recovery during reoxygenation.

Inhibition of succinate dehydrogenase by oxaloacetate has long been recognized [28]–[33] and, as seen from the present work, can be largely prevented by inclusion of rotenone, which is frequently done, but generally without indication that this effect is a goal or is contributing to observed behavior. However, it cannot be simply assumed that rotenone is acting by this mechanism, because it also has large effects on reactive oxygen species production by reverse electron transport from succinate [4]. The present studies show that explicitly considering this process is highly relevant for choosing optimally interpretable conditions to study energization and respiration and for understanding the changes induced by hypoxia/reoxygenation.

Studies of isolated mitochondria have suggested that NEFA-induced uncoupling is via cycling across the membrane involving nonionic diffusion of the protonated form of the fatty acid into the matrix followed by dissociation that delivers a proton to the matrix, then movement of the fatty acid anion out of the matrix on one of the normal anion carriers. Anion carriers that have been implicated include the glutamate:aspartate carrier, the adenine nucleotide translocase, and the uncoupling proteins [26], [27].

We have previously reported that glutamate and the adenine nucleotide translocase inhibitor, carboxyatractyloside, ameliorate deenergization induced by both oleate and H/R in rabbit tubules [15]. The higher sensitivity to oleate in the presence of succinate alone as substrate compared to complex-I substrate combinations including glutamate (Figs. 1 and 5) would seem to further support a role for the glutamate:aspartate carrier, but multiple other lines of evidence from the present studies indicate that glutamate effects on cycling are not the main mechanism. Aspartate did not duplicate the effect of glutamate (Fig. 2). Rotenone had similar effects as glutamate and the two agents were not additive (Fig. 6). Adding malate to generate more oxaloacetate deenergized in a NEFA-dependent fashion and this deenergization was similarly blocked by glutamate and rotenone (Fig. 4 and 5). Inhibition of transamination with AOA prevented and reversed the beneficial effect of glutamate (Fig. 7). Also of importance in this regard, glutamate did not decrease respiration in the presence of oleate as would be expected for an agent that decreased NEFA cycling such as seen for dBSA which clearly acts by the latter mechanism. Instead, glutamate, like rotenone, either increased respiration or preserved it under conditions where it was decaying (Fig. 6). These respiratory effects are most consistent with relief of oxaloacetate-induced respiratory inhibition.

However, some aspects of the observed behavior are not explained solely by relief of oxaloacetate inhibition. In rabbit tubules, when endogenous NEFA were generated by substrate depletion, subsequent addition of glutamate re-energized better than α-ketoglutarate and the difference was abolished by removing the NEFA (Figs. 3D–F). In mouse tubules with exogenous oleate, adding glutamate to α-ketoglutate+malate improved energization and the effect was not entirely blocked by AOA as was the case with adding glutamate to succinate (Fig. 7).

Patterns of energization after H/R in the current studies were similar to those previously reported for rabbit [1], [2], [9], [15], [19] and mouse [11] and expand the earlier data by providing information on the role of oxaloacetate accumulation in succinate-supported energization and the relative effects of different complex-I dependent substrate combinations. Similar to the effects on oleate-treated normoxic tubules (Fig. 6) succinate-supported energization after H/R was improved by both glutamate and rotenone (Fig. 8), which is consistent with oxaloacetate-mediated inhibition. The relative effects were greater in the mouse which is consistent with more oxaloacetate generation resulting from the higher rates of succinate metabolism in the mouse tubules indicated by the respiratory rates of healthy normoxic tubules. However, succinate-supported respiration of the mouse tubules after H/R was no higher than that of rabbit tubules so it is possible that there is also an increased sensitivity to oxaloacetate after H/R.

Support of energization by complex I dependent substrates after H/R differed between mouse and rabbit (Fig. 8) and from the behavior of healthy normoxic tubules treated with oleate (Figs. 1, 5, and 7). In contrast to the better energization seen with complex I-dependent substrates as compared to succinate alone and the equivalent energization relative to succinate+glutamate or rotenone in the healthy normoxic tubules of both species (Figs. 1, 5, and 7), complex I substrates gave weaker energizaton after H/R than succinate plus rotenone in both species and weaker energization than succinate alone and succinate+glutamate in the rabbit (Fig. 9). In the rabbit tubules GM- and AMG-supported energization after H/R was significantly better than AM-supported energization. This was not due to an effect of glutamate on NEFA cycling because it was also seen in the presence of dBSA. In mouse tubules there were no differences between the substrate combinations. Thus, there is no suggestion of effects of substrates on NEFA cycling. The poorer performance of the complex I-dependent substrates may relate to the greater impact of H/R on their ability to support the respiration that compensates for the higher NEFA levels.

Another notable characteristic of the H/R energization data is the poorer recovery of energization of the mouse tubules as compared to the rabbit, which indicates a greater contribution of NEFA-independent factors in that species, particularly considering that the duration of the mouse studies was 30 minutes and the rabbit studies were 67.5 min. These durations of study were chosen based on observations that the energetic deficit in the rabbit tubules was mild at 30 minutes [1], but was fully expressed at that point in the mouse [11] and did not progress much further when the duration of hypoxia was extended to 60 minutes (data not shown).

Studies of respiratory function were part of the original work describing the energetic deficit and showed greater impairment of ADP-stimulated, complex I substrate-dependent respiration than of complex II (succinate)-dependent respiration [2]. However, detailed analysis of the several respiratory states was not done in the prior work and the contribution of NEFA was not known at that time and was not assessed. Insofar as comparable conditions are available for analysis, the current data are remarkably similar to the previous results in showing more severe impairment of complex I-dependent maximal respiration stimulated by ADP and CCCP in both the rabbit and the mouse (Figs. 10–11). The current studies provide substantial new information on additional respiratory conditions and the role of NEFA and, importantly, were done under conditions where measurements of complex II (succinate)-dependent rates were not subject to inhibitory effects of oxaloacetate. Those inhibitory effects did not impact much on respiration in the rabbit tubules, but had a substantial effect in the mouse.

Unlike healthy tubules treated with exogenous oleate, basal respiration after H/R was not stimulated by the high levels of endogenous NEFA. This could be related to the cap on respiratory rates evident in the ADP and CCCP stimulated rates and will limit compensation for the deenergizing effects of NEFA cycling. The presence of NEFA was not required for this respiratory inhibition because it was not alleviated by addition of dBSA to bind them even though dBSA concomitantly improved energization. However, treatment during the reoxygenation period with maneuvers to lower the NEFA burden and increase ATP recovery [2], [8], [10], [11], [15], [23], led to partial repair of the respiratory function defect (Fig. 10C). It is also notable that measurements of respiration provided a more sensitive measure of continuing mitochondrial dysfunction than energization because under both the protected conditions (Fig. 10) and the unprotected conditions tested in the presence of dBSA (Fig. 9), ΔΨ_m_ fully recovered (Figs. 9A and 10A) despite substantial continued respiratory inhibition (Figs. 9C and 10C).

The basis for the greater impairment of complex I by H/R remains unknown. Complex I consists of over 45 proteins [35]–[37] and is more sensitive during injury states by a variety of different incompletely understood mechanisms, including a greater propensity to oxidant damage [35]–[40]. In prior studies we failed to obtain biochemical evidence for defects within complex I itself since NADH coenzyme Q reductase activity was not impaired during the deficit and NADH was not increased [23]. This suggests a defect upstream in the supply of reducing equivalents to it, which could be secondary to changes of TCA cycle enzyme activity or entry of substrates into the mitochondria. The use of permeabilized tubules for measurements of ΔΨ_m_ eliminates entry into cells across the plasma membrane as a consideration and the total substrate concentration was in excess. Additional studies are needed to understand the mechanisms for the respiratory changes that occur. The experiments here that have assessed the contribution of NEFA and highlighted the importance of oxaloacetate in succinate effects, which will help focus the further efforts.

## Conclusions

The present studies have addressed the role of substrate interactions and NEFA in the pathogenesis of the energetic deficit that develops in kidney tubules after H/R, which limits their structural and functional recovery and makes them more prone to processes that cause lethal damage. The data show that assessment of the contribution of succinate-mediated support of energization and respiration must take into account the complicating effects of matrix oxaloacetate production. Improvements of succinate-supported energization and respiration by glutamate are predominantly due to lowering matrix oxaloacetate rather than decreasing uncoupling from transmembrane cycling of NEFA. Mouse tubules have higher rates of succinate utilization and are more affected than rabbit tubules. When effects of oxaloacetate are removed, NEFA added to uninjured tubules at the levels present during hypoxia and ischemia stimulate respiration. In contrast, after H/R respiratory inhibition that is more severe for complex I-dependent substrates contributes to the energetic deficit and impairs respiratory compensation for the increased NEFA. Continued presence of the NEFA is not required for the respiratory inhibition, but lowering NEFA during the prior reoxygenation period and allowing recovery of ATP alleviates it. Our results suggest that maneuvers directed against NEFA during early recovery from hypoxic stress are rational for improving mitochondrial and tubular function.
